# Molecule generation for drug design: A graph learning perspective

**DOI:** 10.1016/j.fmre.2024.11.027

**Published:** 2024-12-20

**Authors:** Nianzu Yang, Huaijin Wu, Kaipeng Zeng, Yang Li, Siyuan Bao, Junchi Yan

**Affiliations:** a School of Artificial Intelligence & Department of Computer Science and Engineering & MoE Lab of AI, Shanghai Jiao Tong University, Shanghai 200240, China; b Shanghai Pinghe School, Shanghai 201208, China

**Keywords:** Drug discovery, Machine learning, Generative models, Graph representation learning, Graph generation

## Abstract

Machine learning, particularly graph learning, is gaining increasing recognition for its transformative impact across various fields. One such promising application is in the realm of molecule design and discovery, notably within the pharmaceutical industry. Our survey offers a comprehensive overview of state-of-the-art methods in molecule design, particularly focusing on *de novo* drug design, which incorporates (deep) graph learning techniques. We categorize these methods into three distinct groups: *i) all-at-once, ii) fragment-based*, and *iii) node-by-node*. Additionally, we introduce some key public datasets and outline the commonly used evaluation metrics for both the generation and optimization of molecules. In the end, we discuss the existing challenges in this field and suggest potential directions for future research.

## Highlights

• This survey offers a comprehensive overview of state-of-the-art methods in molecule design, particularly focusing on 2D *de novo* molecule generation models.
• We categorize these methods into three distinct groups: i) all-at-once, ii) fragment-based, and iii) node-by-node.
• Additionally, we introduce some key public datasets and outline the commonly used evaluation metrics for both the generation and optimization of molecules.
• In the end, we discuss the existing challenges in this field and suggest potential directions for future research.

## 1 Introduction

In recent years, machine learning based drug discovery has been more and more conspicuous since it greatly reduces time, money and labor costs for developing novel drugs [[1], [2], [3]]. In drug development, generating high-quality chemical molecules and optimizing them for desired properties are critical steps. Consequently, the challenge arises in how to apply machine learning methods to effectively generate “good” molecules, with or without additional constraints. Different approaches and models have been designed till now, including those based on variational autoencoder (VAE) [4], generative adversarial networks (GAN) [5], reinforcement learning (RL) [6], etc.

To characterize molecules, several types of molecular representations are devised, ranging from Simplified Molecular Input Line Entry System (SMILES) strings [7] to manually predefined molecular features [8]. Among them, SMILES-based and graph-based representation methods are the most widely used in molecular generation tasks. Early molecular generation methods are SMILES-based. SMILES can be seen as a type of 1D text representation. These SMILES-based methods cannot ensure 100% chemical validity unless complicated constraints are added [9]. Meanwhile, molecules can naturally be represented using graphs, which are essentially a type of 2D representation. Recently, an increasing number of methods have shifted towards graph-based approaches. Unlike the 1D SMILES-based methods, 2D molecule generation approaches can easily ensure that the generated molecules are 100% chemically valid. Additionally, graph-based representation has the ability to accurately depict the inherent structure of molecules. In light of the fact that graph-based representation is currently the mainstream method, this study will specifically concentrate on existing graph-based methodologies.

There also exist surveys on molecule generation and optimization. The work by Xue et al. [10] primarily concentrates on 1D generative methods and does not encompass methods developed in the most recent five years due to its earlier publication date. Guo et al. [11] and Faez et al. [12] both provide a comprehensive overview of the literature in the field of deep generative models for graph generation. But they do not focus on molecules only. Apart from molecules, they also present deep generative models designed for other domains, such as social networks. As for Elton et al. and Alshehri et al. [9,13], they both put emphasis on the four architectures often utilized for molecule design methods. It is also worth mentioning that several studies all involve molecule design methods, based on different molecular representations, including 1D SMILES, 2D representation referring to connectivity graph and 3D representation that contains coordinates of the atoms within a molecule [[9], [11], [12], [13]]. A more detailed discussion on drug representation itself can be found in the review by Guo et al. [14], which specifically focuses on this aspect. In contrast, our review concentrates on molecular generation approaches that utilize 2D-graph representations, with the focus being on the generative methods rather than the details of drug representation. As previously noted, despite the extensive literature on SMILES-based generative models [[15], [16], [17], [18], [19]], these methods have fallen out of mainstream use. Additionally, there is a noticeable disparity in the volume of research between 3D-generative methods for molecules and the more prevalent 2D graph-based methods. In Table 1, we present 1D-, 2D-, and 3D-based molecular representations using a specific molecule as an example. For readers interested in 3D-based methods, existing representative works like G-SchNet [20], E-NF [21], GEN3D [22] and G-SphereNet [23] are recommended.

**Table 1:** **Three different representation methods of a specific molecule**.

Representation Method	Specific Expression
1D	CC(=O)OC1=CC=CC=C1C(=O)O
2D	
3D	

Unlike the existing surveys mentioned above, our review uniquely concentrates on drug design tasks, providing a thorough overview of state-of-the-art molecular design methods specifically from a 2D representation perspective, emphasizing graph learning techniques. Additionally, compared to other surveys and an earlier version of our own survey, we include some more recent methods, particularly some diffusion-based methods, which were not covered in previous surveys.

In this survey, we provide a comprehensive review of the latest graph-based methods for molecule generation and optimization. These methods are classified into three categories based on their generation strategies, reflecting different levels of granularity: *all-at-once, fragment-based*, and *node-by-node*. The survey also covers key public datasets and standard evaluation metrics used in this field. Additionally, we delve into an in-depth analysis of the current challenges faced in this area and proposes three promising directions for future research.

## 2 Preliminaries and problem formulation

### 2.1 Graph-based molecule representation

In the field of graph-based molecule representation [14,24,25], it is common to use a graph G=(V,E) to model a molecule, where V is the graph’s node set mapping to atoms constituting a molecule and E is the graph’s edge set mapping to chemical bonds, with |V|=n and |E|=m. In molecule graph, nodes are sometimes representing atomic types from the periodic table, or representing certain kinds of molecule fragments. The node feature matrix X characterizes the property of each node while the adjacency matrix A characterizes the relationships between each node. Let the number of edge types be b and the number of node types be c, then we have $A\in {\left\{0,1\right\}}^{n\times n\times b}$ and $X\in {\left\{0,1\right\}}^{n\times c}$, where $A_{ijk}=1$ when there exists an edge with type k between the $i^{th}$ and $j^{th}$ nodes, otherwise 0 . We can also represent the molecular graph using the node feature matrix X and the adjacency matrix A, i.e., G=(A,X).

### 2.2 Molecule generation and generation strategies formulation

Generation tasks aim to generate novel samples from a similar distribution as the training data [12]. A molecule generation method intends to generate novel, diverse molecules which follow the unknown data distribution *p*(G) provided by a set of graphs *D_G_*. A machine learning method towards this problem usually proposes a model to learn form large scales of data which either obtains an implicit strategy or estimates the *p*(G) directly and then samples from the distribution to generate new molecules.

**Fig. 1:**
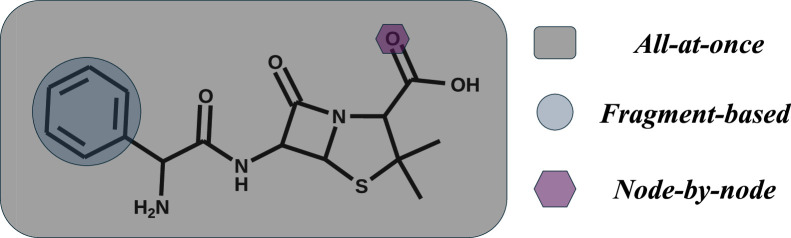
**Comparison of the basic generative units in three molecular generation strategies.** This figure illustrates the fundamental distinction between the three strategies for de novo molecule generation based on their basic generative units: (i) *All-at-once* methods generate the entire molecular graph in a single step; (ii) *Fragment-based* methods use molecular fragments as the building blocks for graph construction; and (iii) *Node-by-node* methods incrementally add individual atoms to build the molecular graph.

To classify existing methods of de novo molecule generation, we propose a classification based on their generation granularity levels, as shown in Fig. 1. Specifically, these methods fall into three categories:

• ***All-at-once:*** The entire molecular graph is generated in a single step, producing the full structure at once. The model takes an input, such as a latent vector, and directly outputs the complete molecular graph without intermediate steps.
• ***Fragment-based:*** This strategy uses molecular fragments as the basic building blocks for generation. The model generates fragments and determines how these fragments should be connected to form the molecular graph. Depending on the specific implementation, the model may generate fragments one at a time or produce multiple fragments simultaneously, as long as fragments serve as the fundamental units for constructing the graph.
• ***Node-by-node:*** This strategy builds the molecular graph atom by atom. At each step, the model generates one atom and decides how to connect it to the existing graph structure. This process repeats iteratively, adding atoms and forming their connections, until the entire molecular graph is complete.

By categorizing methods based on their granularity of generation, this classification highlights the key differences in how molecular graphs are constructed, from generating the entire structure at once to incrementally building it through fragments or individual atoms.

## 3 Methodologies of 2D-based molecule generation

As outlined in Section 2.2, we categorize the existing 2D-based molecule generation methodologies into three groups based on the generation strategies they employ. These methodologies are summarized in Table 2, which provides a clear overview of the different approaches currently utilized.

**Table 2:** **Recent representative works of 2D molecule generation**.

Model	Generation Strategy	Methodology	Venue
VGAE [26]	*all-at-once*	VAE-based	NeurIPS workshop
GraphVAE [27]	*all-at-once*	VAE-based	ICANN
MPGVAE [28]	*all-at-once*	VAE-based	arXiv
Regularized VAE [29]	*all-at-once*	VAE-based	NeurIPS
GDSS [30]	*all-at-once*	Diffusion-based	ICML
CDGS **[**31**]**	*all-at-once*	Diffusion**-**based	AAAI
DiGress [32]	*all-at-once*	Diffusion-based	ICLR
Wave-GD [33]	*all-at-once*	Diffusion-based	NeurIPS
Graph DiT **[**34**]**	*all-at-once*	Diffusion-based	NeurIPS
MolGAN [35]	*all-at-once*	GAN-based	ICML workshop
GraphNVP [36]	*all-at-once*	Flow-based	arXiv
JT-VAE [37]	*fragment-based*	VAE-based	ICML
MHGVAE [38]	*fragment-based*	VAE-based	ICML
HierVAE [39]	*fragment-based*	VAE-based	ICML
MoleculeChef [40]	*fragment-based*	VAE-based	NeurIPS
MoLeR [41]	*fragment-based*	VAE-based	ICLR
PS-VAE [42]	*fragment-based*	VAE-based	NeurIPS
MiCaM [43]	*fragment-based*	VAE-based	ICLR
Modof [44]	*fragment-based*	VAE-based	Nature Machine Intelligence
GCPN [45]	*fragment-based*	RL-based	NeurIPS
DeepGraphMolGen [46]	*fragment-based*	RL-based	Journal of Cheminformatics
RationaleRL [47]	*fragment-based*	RL-based	ICML
FREED [48]	*fragment-based*	RL-based	NeurIPS
GFlowNet [49]	*fragment-based*	GFlowNet-based	NeurIPS
HGLDM [50]	*fragment****-****based*	Diffusion**-**based	CIKM
MARS [51]	*fragment-based*	Sampling-based	ICLR
MIMOSA [52]	*fragment-based*	Sampling-based	AAAI
DEG [53]	*fragment-based*	Sampling-based	ICLR
Mol-CycleGAN [54]	*fragment-based*	GAN-based	Journal of Cheminformatics
CGVAE [55]	*node-by-node*	VAE-based	NeurIPS
SbMolGen [56]	*node-by-node*	VAE-based	Chemical Science
GraphAF [57]	*node-by-node*	Flow-based	ICLR
GraphDF [58]	*node-by-node*	Flow-based	ICML
STGG [59]	*node-by-node*	Spanning-tree-based	ICLR

### 3.1 Generation strategy I: all-at-once

The *all-at-once* deep graph generation approach has emerged as a powerful strategy for generating molecules. This strategy is particularly advantageous for applications requiring high-throughput molecular generation. The molecular generation methods adopting this strategy will be detailed in the following, categorized based on the underlying generation models they are built upon.

#### 3.1.1 VAE-based models

VGAE [26], built upon the variational autoencoder (VAE) framework [4], is a framework designedpervised learning with graph-based data, making it particularly relevant for drug design applications. By leveraging latent variables, VGAE learns interpretable latent representations that can be used to generate new molecular graphs. Unlike VGAE, which can only learn from a single input graph and is limited to modeling a single drug pattern, GraphVAE [27] is a VAE-based generative model capable of handling a set of molecular graphs, making it particularly valuable in drug design. The encoder of GraphVAE employs a graph convolutional network (GCN) [60] to embed input molecular structures into a continuous latent representation **z**. The decoder then generates a probabilistic fully-connected graph, from which discrete molecular samples can be drawn. However, GraphVAE faces challenges in aligning effectively with the training data distribution and requires a costly graph matching procedure, which hinders its efficiency in practical drug design applications. In order to address these issues, MPGVAE [28] integrates a message passing neural network (MPNN) [61] to the encoder and decoder, enhancing the efficiency of both encoding and decoding processes. Furthermore, Ma et al. [29] propose a specialized regularization framework for training VAEs that encourages the satisfaction of validity constraints for molecules, i.e., the number of bonding-electron pairs must not exceed the valence of an atom.

#### 3.1.2 Diffusion-based models

**Fig. 2:**
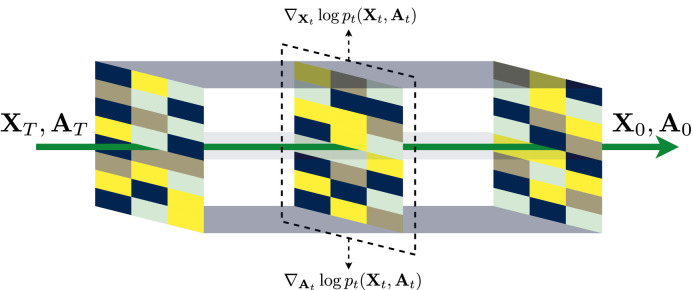
**Reverse-time diffusion process of GDSS**.

GDSS [30] presents an innovative graph diffusion process aimed at modeling the intricate relationships between atoms and bonds within molecular structures. This approach employs a system of stochastic differential equations (SDEs) to capture the joint distribution of these molecular components. By formulating specialized score matching objectives, GDSS effectively estimates the gradient of the joint log density for each atom and bond, facilitating a deeper understanding of molecular interactions. To streamline the process of molecular generation, GDSS introduces a novel solver for the SDE system, enhancing the efficiency of sampling from the reverse diffusion process. This is illustrated in Fig. 2, which showcases the reverse-time diffusion dynamics employed in the context of drug design. Moreover, it introduces a novel solver for the SDE system to facilitate efficient sampling from the reverse diffusion process. We demonstrate the reverse-time diffusion process of GDSS in Fig. 2. However, GDSS encounters limitations in both generation quality and sampling speed. It shows a clear gap between the generated molecular data and true distributions, necessitating additional Langevin correction steps to mitigate approximation errors. These corrections increase computational costs and extend inference times, highlighting the limitations of the graph score estimation model and hindering the practical use of GDSS in generating molecular graphs for drug discovery.

To address the limitations of GDSS, CDGS [31] introduces a conditional diffusion framework that enhances both sampling speed and model accuracy in drug design. Specifically, CDGS employs a forward diffusion process guided by stochastic differential equations (SDEs) to navigate the relationships between atoms and bonds, treating molecular structures and their properties as conditional elements during the reverse generation phase. A hybrid noise prediction model is utilized to capture both global molecular architectures and local atom-bond interactions from intermediate molecular representations. To boost sampling efficiency, CDGS integrates ordinary differential equation (ODE) solvers that exploit the semi-linear characteristics of probability flow ODEs, facilitating similarity-constrained optimization of molecules through gradient-guided adjustments.

Moreover, it is important to note that GDSS and CDGS project molecular graphs into a continuous space, introducing Gaussian noise to atom features and bond connections. However, this approach removes the inherent sparsity of molecular graphs, resulting in entirely noisy graphs where structural information, such as connectivity or cycle counts, remains undefined. Consequently, the continuous diffusion process can present challenges for the denoising network in capturing the structural properties of the data. In contrast, inspired by D3PM [62], a new method DiGress [32] implements a discrete diffusion process that systematically adjusts molecular graphs by adding or removing bonds and altering atom types. Additionally, the conditioning on graph-level properties enhances the relevance of generated molecules to specific therapeutic targets, making DiGress a powerful tool for optimizing drug design processes and improving the efficiency of the drug discovery pipeline.

While DiGress has made notable advancements compared to GDSS, it still faces challenges in accurately estimating the joint distribution of measurements derived from node features and molecular graph structures. This challenge primarily arises from the approach of deriving separate embeddings for atoms and bonds, treating them as distinct entities. Therefore, a more recent diffusion-based method called Wave-GD [33] has been introduced. Wave-GD harnesses the spectral dependencies between atom and bond signals to more effectively characterize their joint distributions through a score-based diffusion model. By capturing their multi-resolution coherence, this model demonstrates the capability to generate high-fidelity molecules while preserving the frequency characteristics observed in the training molecular samples.

Previous diffusion-based methods in molecular generation have struggled to integrate multiple property constraints, such as synthetic score and gas permeability, within a single diffusion model framework. Addressing this gap, the Graph Diffusion Transformer (Graph DiT) [34] enables multi-conditional molecular generation by encoding diverse property constraints directly into the diffusion process. Graph DiT captures numerical and categorical property features through a dedicated condition encoder, using clustering for numerical properties and one-hot encoding for categorical ones. The model further introduces a unique graph-dependent noise model that applies noise collectively to atom-bond relationships, enhancing the accuracy of noise estimation. Additionally, adaptive layer normalization (AdaLN) within the Transformer-based denoiser adjusts molecular statistics to align with each condition. Together, these innovations allow Graph DiT to generate molecules that meet multiple property criteria, demonstrating its utility in complex tasks such as polymer inverse design.

#### 3.1.3 Others

**Fig. 3:**
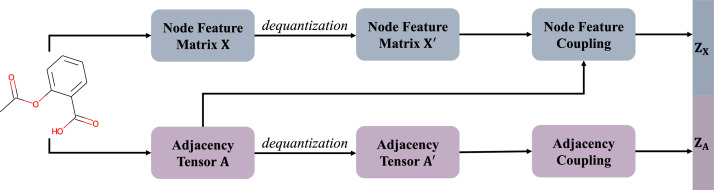
**Forward transformation of GraphNVP**.

In addition to the aforementioned VAE-based and diffusion-based methods, there are also approaches that utilize other generative models, such as MolGAN [35] and GraphNVP [36]. MolGAN proposes an implicit generative model for molecular graphs, which adapts generative adversarial networks (GAN) [5] for graph-structured data and integrates a reinforcement learning objective to encourage the generation of molecules with desired properties. GraphNVP is the first known molecule generation model based on invertible normalizing flow [63,64]. As shown in Fig. 3, it performs *dequantization* technique [63,64] to transform discrete adjacency tensor **A** and node label matrix **X** into continuous variables and then uses coupling layers to obtain latent representations **z_A_***,* **z_X_. z_A_** and **z_X_** are concatenated together to obtain the final latent representation **z** of the molecule, i.e., **z** = CONCAT(**z_A_***,* **z_X_**). After sampling a latent vector **z** from a known prior distribution and splitting **z** into **z_A_** and **z_X_**, GraphNVP takes two steps to generate a molecule. The first is generating a molecular graph structure **A** from **z_A_** and the second is generating atom attributes **X** based on the structure **A** from **z_X_**. Furthermore, GraphNVP trains a linear regressor on the latent space of molecules to quantitatively estimate a target chemical property. This allows for the interpolation of the latent vector of a randomly selected molecule in the direction of the desired property, as determined by linear regression, thereby significantly enhancing the optimization of molecular designs.

### 3.2 Generation strategy II: fragment-based

The *fragment-based* approach has gained considerable traction for molecular generation, leveraging rational substructures, or fragments, as building blocks to construct complex molecules. This strategy is particularly beneficial in applications where preserving functional group integrity is crucial.

By assembling molecules from well-defined fragments, this strategy enables more efficient exploration of chemical space and has been widely adopted in recent research to generate high-quality molecules. In the following, we will delve into the various methods that apply this strategy, organized according to the different generation models they employ.

#### 3.2.1 VAE-based models

**Fig. 4:**
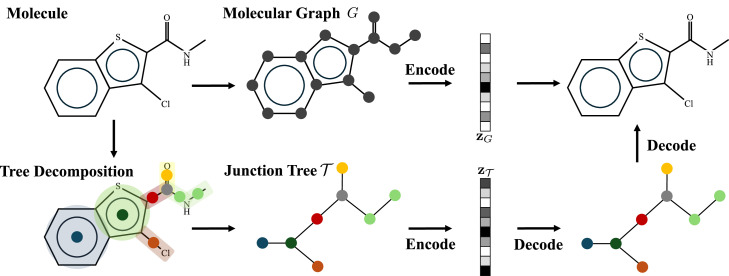
**Overview of JT-VAE**.

Among these methods, a considerable number are based on the variational autoencoder. An earlier typical work proposes a model named JT-VAE [37] which first decomposes the molecular graph G into its junction tree, where each node in the tree represents a substructure of the molecule. JT-VAE then encodes both the junction tree and molecular graph into their latent embeddings **z**_T_ and **z***_G_*, respectively. As for the decoding phase, JT-VAE first reconstructs the junction tree from **z**_T_ then generates molecule graph from the predicted junction tree by a graph decoder which learns how to assemble subgraphs. The JT-VAE pipeline, as previously described, is presented in Fig. 4. JT-VAE achieved an impressive 100% validity by utilizing a junction tree representation, which offers significant advantages in drug design scenarios. This method streamlines the process, as constructing a tree structure is inherently less complex than generating a general molecular graph with degree constraints, making it easier to explore and optimize molecular designs efficiently.

MHG-VAE [38] addresses the problem of producing invalid molecules, known as the *decoding error issue*, by leveraging a molecular hypergraph grammar (MHG). Unlike traditional methods that struggle with atom-level connections and stereochemistry, MHG-VAE simplifies the process by avoiding the need for auxiliary neural networks. In this approach, atoms are represented as hyperedges, and bonds as nodes, allowing for precise connections that respect atomic valency. This innovative framework ensures the generation of valid molecules while effectively incorporating stereochemical information, making it particularly advantageous in drug design scenarios.

HierVAE [39], another VAE-based model designed by the same authors as JT-VAE, introduces larger and more flexible graph motifs as building blocks, exhibiting enhanced performance when dealing with larger molecules. The encoder of HierVAE generates a multi-resolution representation for each molecule, progressing in a fine-to-coarse fashion from atoms to connected motifs. Each hierarchical level in this model integrates the encoding of its lower-level constituents with the graph structure at that level. The autoregressive coarse-to-fine decoder of HierVAE adds motifs sequentially, one at a time. This process interweaves the selection of a new motif with the task of determining how it connects to the evolving molecular structure.

In the realm of drug discovery, there’s often a necessity for a specific scaffold to be included in the synthesized molecule. MoLeR [41], also based on VAE, has been developed to cater to this need, facilitating the extension of partial molecules. MoLeR integrates motifs (molecule fragments) into its atom-by-atom generation process. When dealing with atoms that are part of a motif, MoLeR concatenates the initial chemically relevant features with the motif embedding. For atoms not associated with a motif, a special embedding vector is employed to indicate the absence of a motif. To define a concrete generation sequence, MoLeR initially opts for an initial atom selection. Subsequently, for each partial molecule, it proceeds to select the next atom from those that are adjacent to the atoms previously generated. Following each selection, in cases where the presently chosen atom is part of a motif, MoLeR incorporates the entire motif into the partial graph at once. Furthermore, MoLeR enhances its molecular generation process by integrating Molecular Swarm Optimization (MSO) [65], which aids in the optimization of the molecular structures.

Previous methods like JT-VAE and HierVAE constructed the vocabulary of molecular fragments using simple hand-crafted rules, which may not effectively reveal frequent patterns in chemical datasets. Moreover, in fragment-based methods, molecular generation and assembly occur either autoregressively, as seen in HierVAE, or follow a pre-defined tree structure, as in JT-VAE. Both strategies exhibit inherent limitations: each newly generated molecular fragment is restricted to attaching only to a local set of previously generated fragments, resulting in a lack of flexibility. This constraint can hinder the exploration of diverse molecular structures crucial for drug discovery. To overcome these issues, PS-VAE [42] has been developed. PS-VAE begins by creating a vocabulary of molecular fragments from a given dataset, starting with distinct atoms and progressively merging neighboring fragments to update the vocabulary. This merge-and-update strategy leads to the formation of *principal subgraphs*, a novel concept introduced in this paper, which represents frequent and significant repetitive patterns in molecules. PS-VAE also theoretically ensures that any *principal subgraph* can be covered by the developed vocabulary. Additionally, PS-VAE introduces a two-step subgraph assembling strategy: it initially predicts a set of molecular fragments sequentially and subsequently performs a global assembly of all generated subgraphs. This method reduces dependency on permutations and places greater emphasis on global connectivity, providing a more robust approach for molecular assembly compared to traditional fragment-based methods in drug design.

In the original paper of MiCaM [43], another method based on VAE, the authors also highlight the crucial importance of developing an effective motif vocabulary. Accordingly, MiCaM devises an algorithm that identifies the most prevalent substructures based on their frequency within the molecule library. It scans the entire library to detect fragment pairs that frequently occur adjacent to each other within molecular graphs and subsequently merges these pairs into larger fragments. This merging process is repeated for a pre-determined number of steps, accumulating fragments to form a comprehensive motif vocabulary. The obtained motifs retain their structural connectivity information. Therefore, they are referred to as *connection-aware motifs* in the paper. The generator of MiCaM operates by concurrently selecting motifs to be added and specifying their connection modes. During each step of the generation process, MiCaM concentrates on a nonterminal connection site within the current generated molecule. This site is then utilized to identify another connection, presenting two alternatives: (1) opting for a connection from the motif vocabulary, indicating the addition of a new motif, or (2) choosing a connection from the current molecular structure, a move that leads to the cyclization of the molecule.

Modof [44] stands as another advanced deep generative method designed for molecule optimization. It operates by predicting a single disconnection site within a molecule and subsequently modifying the molecule by altering the fragments at that site, including elements such as ring systems, linkers, and side chains. What sets Modof apart from existing molecule optimization methods is its approach to learning from and encoding the disparities between molecules before and after optimization at a single disconnection site. When it comes to modifying a molecule, Modof generates only one fragment that represents the anticipated difference. This is achieved by decoding a sample obtained from the latent *difference* space. Subsequently, Modof removes the original fragment at the disconnection site and replaces it with the newly generated fragment. Its model training also employs the VAE objective.

MoleculeChef [40] addresses a critical issue in previous molecular generation methods: the lack of guidance on synthesizing generated molecules. This limitation raises concerns about the practical synthesize-ability of these molecules in real laboratory settings. In contrast, MoleculeChef generates novel molecules by simulating virtual chemical reactions, closely mimicking laboratory discovery processes. It maps a multiset of reactants to a probability distribution in latent space and sequentially generates these reactants using a recurrent neural network (RNN) [66]. The generated reactants are then processed through a reaction predictor, implemented using the Molecular Transformer [67]. To overcome training challenges associated with traditional VAE objectives, MoleculeChef employs the Wasserstein autoencoder (WAE) objective [68], enhancing its capability to generate synthesizable molecules.

#### 3.2.2 RL-based models

There also exist several works using reinforcement learning (RL) to optimize the properties of generated molecules. An earlier work GCPN [45] represents a notable goal-directed approach to 2D molecule generation in drug design. Unlike traditional graph generative models that struggle to incorporate specific molecular properties or constraints, GCPN leverages RL to effectively represent these requirements through tailored environment dynamics and reward functions. This allows for active exploration of molecular space beyond existing dataset samples, overcoming the limitations faced by generative models. GCPN formulates the molecule construction as a Markov Decision Process (MDP), building molecules sequentially by either adding bonds between existing atoms or integrating new fragments. The approach utilizes intermediate and final rewards to guide generation, incorporating both domain-specific and adversarial rewards to optimize the synthesized molecules for desired properties. DeepGraphMolGen [46] builds upon the foundation of GCPN and takes a further step in addressing the challenge of generating novel molecules with desired interaction properties. In DeepGraphMolGen, interaction binding models are learned from binding data using GCNs. Recognizing that experimentally obtained property scores may contain potentially significant errors, DeepGraphMolGen incorporates a robust loss for the model. Notably, in contrast to GCPN, the final reward in DeepGraphMolGen includes the pKi value [69] of the final molecule as predicted by the trained model.

**Fig. 5:**
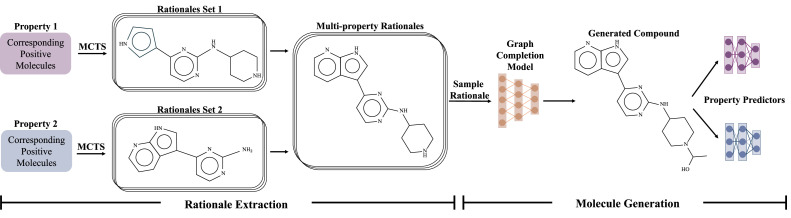
**Overview of RationaleRL**.

While GCPN and DeepGraphMolGen are capable of generating molecules with desired properties, it remains challenging for them to generate molecules that simultaneously satisfy many property constraints. RationaleRL [47], as depicted in Fig 5, an RL-based 2D molecule generation method, has been introduced to specifically tackle this limitation. Its core idea involves composing molecules from a vocabulary of substructures known as *molecular rationales*. The first step of RationaleRL is extracting rationales that are likely accountable for each property from molecules by Monte Carlo Tree Search (MCTS) [70] and combining them for multiple properties. Specifically, during the search process, each state in the search tree means a subgraph of the molecule, and the property score of the subgraph indicates the reward. Then RationaleRL uses graph generative models to expand the rationales into full molecules. To generate realistic compounds, the graph generator is trained in two phases, namely pre-training phase and fine-tuning phase. After pre-training on a large set of real molecules, the graph generator is fine-tuned on property-specific rationales through multiple iterations using policy gradient.

#### 3.2.3 Others

Previous RL-based methods for goal-directed molecular design often emphasized relatively straightforward objectives, such as QED [71]. However, achieving high scores in these simple molecular properties does not necessarily ensure drug-likeness or therapeutic potential, underscoring the importance of adopting more relevant design objectives in generative tasks. In contrast, another RL-based approach known as FREED [48] places its focus on a more meaningful optimization target, namely, the docking score. This choice is made because docking simulations [72,73] provide a more direct and practical proxy for assessing therapeutic potential, making it a valuable metric in the context of molecular design. FREED adopts a strategy for generating molecules that involves attaching a chemically realistic and pharmacochemically acceptable fragment unit to a given sub-graph at each step. Importantly, the model is enforced to create new bonds only at attachment sites that are considered suitable based on the fragment library preparation step. These strategies effectively leverage prior knowledge in medicinal chemistry, ensuring that molecule generation remains confined within the chemical space conducive to drug design. Additionally, FREED explores various explorative algorithms, incorporating curiosity-driven learning and prioritized experience replay (PER) [74]. In particular, FREED introduces an innovative PER method that defines priority based on the novelty of experiences, estimated by the predictive error or uncertainty of the auxiliary reward predictor’s outcome. This approach aims to enhance the robustness of molecular generation, addressing the shortcomings of previous methods and fostering the exploration of diverse solutions, which is crucial for advancing drug design and discovering innovative therapeutics.

A novel generative approach called GFlowNet [49] has recently emerged, closely linked to reinforcement learning (RL) and particularly relevant for drug design. GFlowNet addresses the challenge of learning a stochastic policy for generating molecular graphs through a sequence of actions, ensuring that the likelihood of generating a molecule is directly proportional to a specified positive reward. By conceptualizing the generative process as a flow network, GFlowNet effectively manages multiple pathways that can lead to the same molecular structure, allowing for flexible exploration in molecular assembly. In this context, nodes represent molecular states, while edges correspond to actions such as adding fragments from a predefined vocabulary or concluding the generation process. This framework facilitates the generation of diverse and potentially novel molecules, which is essential for advancing drug discovery efforts.

In our previous discussion of *all-at-once* approaches, we have introduced several diffusion-based methods. Within the *fragment-based category*, there is also a diffusion-based method: the Hierarchical Graph Latent Diffusion Model (HGLDM) [50]. HGLDM adopts a latent diffusion framework and leverages PS-VAE as its molecular autoencoder. Since PS-VAE [42] itself is a *fragment-based approach*, HGLDM is inherently *fragment-based* as well. Previous *all-at-once* diffusion-based methods often use transition matrices to corrupt discrete data or relax the data into continuous space, which complicates extensible conditional generation—such as adapting to text-based inputs—and increases computational demands, particularly when diffusing bond type matrices. HGLDM overcomes these limitations by operating in a latent space, effectively reducing computational overhead while supporting flexible conditional generation. Unlike its simpler variant, the Graph Latent Diffusion Model (GLDM), HGLDM incorporates both graph-level and fragment-level embeddings, enabling it to capture nuanced relationships between molecular structure and properties.

Another emerging line considers molecule generation as a sampling procedure. One noteworthy method in this category is MARS [51]. The core concept of MARS involves initiating the process from a seed molecule and continually generating candidate molecules by making modifications to fragments of molecular graphs from previous iterations. In MARS, the task of molecular design is framed as an iterative editing process, with the overall objective being composed of multiple property scores. To search for optimal chemical compounds, MARS employs the annealed Markov Chain Monte Carlo (MCMC) sampling technique [75]. This approach allows for the exploration of chemicals with novel and diverse fragments. MARS utilizes graph neural networks (GNNs) to represent proposals for modifying molecular fragments. These GNNs adaptively learn their parameters to propose fragment modifications. While MPNNs are used in practice, other GNN architectures can also be integrated into the framework. Moreover, MARS leverages the sample paths generated on-the-fly to adaptively train the proposal network, eliminating the need for external annotated data. This adaptive learnable proposal mechanism enables MARS to continuously enhance the quality of molecule generation throughout the process.

MIMOSA [52] is another molecule generation approach built on the MCMC sampling method. MIMOSA unfolds in three distinct stages. Initially, MIMOSA focuses on training a pair of property-neutral GNNs. These networks are tasked with the prediction of molecular topologies and substructure types. These substructures encompass atoms and rings. This step is crucial for improving the embeddings of molecules, aiding in the later stage of sampling.

Subsequently, MIMOSA leverages these predictions to conduct three core substructure manipulations: ADDITION, REPLACEMENT, and REMOVAL to generate new molecule candidates. In its final stage, MIMOSA evaluates these newly generated molecules, assigning them weights based on criteria such as structural resemblance and drug property constraints. Molecules that fulfill these specified criteria are then chosen for additional processing rounds.

There’s another fragment-based method called DEG [53], which is also related to sampling. This method addresses a key challenge in drug design: the reliance on deep neural networks that require extensive training on large datasets, often containing tens of thousands of examples. However, real-world drug discovery typically involves limited, class-specific chemical datasets, sometimes just a few dozen samples due to the labor-intensive nature of experimentation. This scarcity hampers the ability of deep learning models to effectively explore the diverse molecular landscape needed for viable drug candidates. In response, DEG emerges as a generative model optimized for data efficiency, capable of learning from much smaller datasets than traditional models. Utilizing a learnable graph grammar, DEG generates molecules through production rules derived directly from training data, without manual intervention. The model also incorporates grammar optimization to enhance chemical insights. With training that leverages Monte Carlo (MC) sampling [76] and the REINFORCE algorithm [77], DEG facilitates robust learning in data-scarce environments, significantly advancing drug design by enabling the generation of novel, synthesizable molecules despite limited data.

Additionally, it’s noteworthy to mention a GAN-based method designed for molecular optimization, Mol-CycleGAN [54]. More specifically, it employs the CycleGAN [78] architecture in the context of drug design. A key strength of Mol-CycleGAN lies in its capacity to discern and learn transformation rules from compound sets, based on their desired and undesired property values. It functions within a latent space, which is trained by another model. Specifically, in the case of Mol-CycleGAN, this latent space is derived from the JT-VAE model we discussed earlier. The capability of Mol-CycleGAN to generate molecules with particular desired properties is well-demonstrated, particularly in terms of structural and physicochemical attributes. Notably, the molecules produced by this model closely resemble their initial forms, with a tunable degree of similarity, adjustable through a designated hyperparameter.

### 3.3 Generation strategy III: node-by-node

In addition to synthesizing molecules directly or employing substructures as foundational building blocks, recent advancements have introduced alternative strategy for molecular generation. These innovative approaches, known as *node-by-node*, offer a highly granular method for constructing molecules atom by atom. This fine-grained control provides significant flexibility in molecular design, making it particularly useful in applications where precise atom-level modifications are required. As a result, the *node-by-node* strategy has garnered attention in recent research for the potential to generate highly customizable molecular structures. Up next, we will present an overview of the methods employing this strategy, classified by the generation models they are based on.

#### 3.3.1 VAE-based models

CGVAE [55] is a VAE-based generative model tailored for drug design, utilizing gated graph neural networks (GGNN) in its architecture [79]. In this framework, molecules are represented as graphs, with atoms as nodes and bonds as edges. The model embeds each atom into a latent vector, which is sampled from a normal distribution. The decoder initializes the atoms with these latent variables and sequentially generates bonds using two decision functions: FOCUS, which selects the atom to visit, and EXPAND, which determines which bonds to add. The process continues until a stopping criterion is reached. To ensure chemical validity, the EXPAND function applies valency masking. CGVAE optimizes the generated molecular structures based on specific numerical properties, enabling the efficient creation of viable and synthesizable compounds, thereby enhancing the drug discovery process.

Lim et al. [56] propose another VAE-based method that is able to generate molecules with target properties while maintaining an arbitrary input scaffold as a substructure. In our survey, we refer to their method as SbMolGen. Its encoder adopts a variant of interaction network [80,61] to encode one complete molecule graph G into a latent vector **z**, from which the decoder is trained to recover molecules. Specifically, the decoder takes a scaffold *S* as input and sequentially adds nodes and edges to *S* based on three loop stages namely NODE ADDITION, EDGE ADDITION, NODE SELECTION and an extra final stage named ISOMER SELECTION. Furthermore, we can concatenate the whole-molecule properties vector and scaffold properties vector with **z** sampled from latent space to condition the decoding process.

#### 3.3.2 Flow-based models

We have previously introduced two VAE-based methods for molecule generation in a node-by-node fashion. Additionally, there are flow-based methods available for generating molecules. GraphAF [57] is a representative flow-based model, whose concept is actually similar to the previously introduced GCPN. Both formulate the problem of molecular graph generation as a sequential decision process. Specifically, beginning with an empty graph, GraphAF sequentially generates a new atom at each step, which is based on the structure of the current sub-graph. Subsequently, the bonds connecting this newly added atoms with the existing ones are systematically formed, taking into account the existing molecular graph structure. This iterative process continues until the generation of all atoms and bonds is complete. The core concept of GraphAF involves defining an invertible transformation from a base distribution (like a multivariate Gaussian) to a molecular graph structure G = (*A, X*). In every round of generation, GraphAF, given the existing sub-graph structure, employs a stack of multiple layers of a modified version of Relational GCN [81] to derive the embeddings for each atom. Following this, a sum-pooling operation is applied to these node embeddings to obtain the embedding of the entire sub-graph. This sub-graph embedding is then set as the mean and standard deviations of Gaussian distributions, which are subsequently used to generate the nodes and edges. Additionally, GraphAF suggests that the molecule generation process can be refined through reinforcement learning, aiming to optimize the properties of the molecules generated.

It’s important to recognize that, akin to GraphNVP [36], GraphAF converts discrete molecular samples into continuous data through a technique called *dequantization* by adding real-valued noise. However, this *dequantization* process limits the model’s ability to accurately represent the original discrete distribution of molecular structures, posing a challenge in training and hindering the accurate capture of the true distribution. This can lead to a wide variety of generated molecules that may not reflect viable drug candidates. To address these challenges, GraphDF [58] builds upon GraphAF by generating molecular graphs using discrete latent variables, which are sampled from multinomial distributions. This approach allows for a more accurate representation of molecular structures. In GraphDF, the generation process mirrors that of GraphAF but utilizes discrete flow models to map latent variables to new nodes and edges. By employing discrete latent variables, GraphDF enhances the reliability of generated molecules, making it a promising tool in drug discovery.

#### 3.3.3 Others

STGG [59] stands out from prior VAE-based and flow-based methods as the first framework to utilize a spanning tree-based approach for molecular graph generation. This unique methodology conceptualizes the generation of molecular graphs through the construction of a spanning tree along with residual edges. This approach leverages the inherent sparsity found in molecular graphs, enabling the use of efficient tree-constructive operations to establish molecular graph connectivity. STGG ensures that the generated molecular graphs adhere to chemical valence rules by applying constraints based on the intermediate graph structure formed during the construction process. Additionally, STGG introduces an innovative Transformer architecture [82], incorporating tree-based relative positional encodings, to effectively facilitate the tree construction procedure.

### 3.4 Molecular optimization

While high-quality unconditional generation is a prerequisite, the ability to condition generation on graph-level properties is crucial for many applications. In the previous three subsections, we classified and introduced existing 2D molecule generation methods based on different generation strategies. During these discussions, we also briefly touched upon how some methods optimize molecular properties during generation, a process known as molecular optimization.

Molecular optimization is a specialized field of study. For instance, some papers dedicated to this topic focus on modifying a given reference molecule to enhance desired properties, which differs from our focus on *de novo* molecule generation. Although this survey primarily reviews existing 2D molecule generation methods and does not emphasize optimization, we have chosen to introduce it here briefly. We recommend the survey by Xia et al. [83] for readers interested in molecular optimization.

Existing molecular optimization techniques can be broadly categorized into constrained generative models (CGM) and combinatorial optimization (CO). CGMs utilize deep generative networks, including VAE [55,84,85,19], GAN [86], flow-based models [57] and diffusion models [87], to model molecular distributions. These networks project molecules into a latent space for optimization, followed by reconstructing the optimized molecules. However, achieving an ideal smooth and discriminative latent space, essential for CGMs, poses a substantial practical challenge [88,89].

Conversely, CO-based methods search for desired molecules directly within the explicit discrete space. This category encompasses techniques such as reinforcement learning [45,90,91,47], evolutionary algorithms [92], Markov Chain Monte Carlo [51,52], tree search [93], and bayesian optimization [94,95]. Despite their effectiveness, CO algorithms require numerous oracle calls, resulting in computational inefficiency during inference.

## 4 Comparisons of three generation strategies

The generation at different granularities can have different advantages in specific applications. In general, the *all-at-once* scheme can be highly efficient, generating entire molecules in a single step. This approach is advantageous in terms of speed and computational resources, making it suitable for high-throughput applications. However, it may sometimes lack the flexibility needed for incremental generation and optimization. Additionally, *all-at-once* methods often struggle more with ensuring the validity of the generated molecules compared to *node-by-node* and *fragment-based* strategies.

The *fragment-based* strategy allows the editing of sub-structures of a molecule. This can be particularly meaningful for specific functionalities and reactions, as certain fragments or motifs within a molecule are often responsible for its chemical properties. This method can be more efficient than node-by-node generation and better at preserving the integrity of functional groups, but it may still lack the flexibility to generate entirely novel structures without predefined fragments.

Finally, the *node-by-node* strategy is flexible, allowing for detailed modifications of individual atoms or bonds. However, this approach can be less efficient for generation due to its step-by-step nature and may have difficulty modeling higher-level (sub)structure information, leading to potential inconsistencies in larger or more complex molecules.

Furthermore, we have identified a significant trend in molecule generation methods that aligns with advancements in generative models. Initially, there was a preference for using VAEs. However, with the rising popularity of diffusion models, recent works have adopted these newer models for molecule generation, as they have shown superior performance in tasks such as image generation [96,97]. Recent diffusion-based molecule generation methods typically employ an all-at-once strategy. Unlike VAEs, which are flexible and can be applied across three different strategies, integrating diffusion models with the other two strategies is non-trivial and requires further exploration. Additionally, incorporating valency constraints during the denoising process is challenging, resulting in diffusion-based molecule generation methods not yet achieving 100% validity in the generated molecules. This issue is worth exploring further in future research.

## 5 Datasets and evaluation metrics

The success of molecule generation models relies on both the quality of the datasets used for training and the evaluation metrics used to assess their performance. High-quality datasets ensure that models can generalize across different chemical spaces, while robust evaluation metrics are essential for determining the practical utility of the generated molecules. In this section, we provide an overview of the key datasets and evaluation metrics commonly employed in molecule generation research.

***Datasets****.* We present a compilation of prominent publicly accessible datasets, commonly employed in molecule generation and optimization endeavors, as summarized in Table 3.

**Table 3:** **Representative datasets for molecule generation**.

Dataset	Description	Number of molecules	Link
ChEMBL	Bioactive molecules with drug-like properties	*>* 2*,* 000*,* 000	https://www.ebi.ac.uk/chembl/
DrugBank	FDA-approved drugs and other drugs public available	*>* 14*,* 000	https://www.drugbank.ca/
GDB-17	Enumeration of small organic molecules up to 17 atoms	*>* 166*,* 000*,* 000*,* 000	http://gdb.unibe.ch/downloads/
ZINC15	Commercially available compounds	*>* 750*,* 000*,* 000	http://zinc15.docking.org/
QM9	Stable small organic molecules made up of CHONF atoms	133*,* 885	http://quantum-machine.org/datasets/
GEOM	contains molecule conformers with experimental data.	430*,* 000	https://github.com/learningmatter-mit/geom
PubChemQC	Compounds with quantum chemistry estimated property based on density functional theory	3*,* 981*,* 230	http://pubchemqc.riken.jp/

***Evaluation Metrics****.* We commonly use the following metrics to evaluate the effectiveness of molecular generation methods:

• ***Validity***: This metric measures the percentage of generated molecules that are chemically valid, meaning they adhere to the rules of chemistry, which ensures that the generated structures are feasible.
• ***Novelty***: This metric assesses the fraction of generated molecules that do not appear in the training data. A higher novelty score indicates that the model can generate new, previously unseen molecules, which is crucial for discovering new compounds.
• ***Diversity***: This metric measures the pairwise molecular distance among generated molecules. High diversity ensures that the generated molecules cover abroad range of chemical space, which is important for exploring various potential candidates.
• ***Uniqueness***: This metric measures the ratio of unique molecules among the generated set. This metric ensures that the model is not generating the same molecule multiple times, promoting a variety of different structures.
• ***Reconstruction***: This metric evaluates the percentage of molecules that can be accurately reconstructed from their latent variables. It assesses the model's ability to learn and reproduce the underlying data distribution.

Furthermore, in the process of molecular generation, numerous studies also consider optimizing the properties of the generated molecules, such as quantitative estimate of drug-likeness (**QED**) [71], synthetic accessibility (**SA**) [98], octanol-water partition coefficients (**logP**) [99], and so on. Specifically, SA represents the difficulty of drug synthesis, with higher values indi cating easier synthesis as it is normalized between 0 and 1. QED quantifies the likelihood of a molecule being a potential drug candidate. LogP indicates the molecule’s partition coefficient between octanol and water, where logP values between −0.4 and 5.6 are considered favorable for drug candidates [100]. These metrics provide a comprehensive framework for evaluating molecular generative models, enabling assessment of their ability to generate chemically valid, diverse molecules with desirable properties.

To provide a clearer benchmark for the performance of different molecular generation methods, we summarize the results reported in existing published works in Table 4. As this review introduces a wide range of methodologies, not all methods are included in the table, as many do not report results on the same datasets or evaluation metrics. We select two datasets, ZINC250k and QM9, as they provide the most comprehensive benchmarks for the methods reviewed. ZINC250k contains approximately 250,000 drug-like molecules extracted from the ZINC15 database, which we introduced earlier as a widely used source for molecular data. QM9, on the other hand, is a dataset of small organic molecules with up to nine heavy atoms. For evaluation, we focused on three commonly used metrics—*Validity, Uniqueness*, and *Novelty*—since these metrics are the most consistently reported across studies. It is important to note that not all methods included in the table provide results for every dataset or metric, even if they have been evaluated on one of the selected datasets.

**Table 4:** **Benchmarking the performance of molecular generation methods on the ZINC250k and QM9 datasets**.

Category	Method	Architecture	ZINC250k			QM9		
			*Validity*	*Uniqueness*	*Novelty*	*Validity*	*Uniqueness*	*Novelty*
*All-at-once*	VGAE [26]	VAE-based	7.0	–	100.0	81.0	24.0	61.0
	GraphVAE [27]	VAE-based	–	–	–	52.0	58.3	61.3
	MPGVAE [28]	VAE-based	–	–	–	91.0	68.0	54.0
	Regularized VAE [29]	VAE-based	35.0	–	100.0	86.6	–	97.5
	GDSS [30]	Diffusion-based	100.0	99.6	100.0	100.0	98.5	86.3
	CDGS [31]	Diffusion-based	100.0	99.9	99.9	100.0	96.8	69.6
	DiGress [32]	Diffusion-based	–	–	–	99.0	96.2	–
	Wave-GD [33]	Diffusion-based	–	–	–	97.0	98.9	89.8
	MolGAN [35]	GAN-based	–	–	–	98.0	10.0	94.0
	GraphNVP [36]	Flow-based	–	–	–	83.1	99.2	–
*Fragment-based*	JT-VAE [37]	VAE-based	100.0	98.8	98.8	100.0	54.9	38.6
	MHGVAE [38]	VAE-based	100.0	–	–	–	–	–
	HierVAE [39]	VAE-based	–	13.1	13.1	–	41.6	28.5
	MoLeR [41]	VAE-based	100.0	99.6	99.3	100.0	94.0	35.5
	PS-VAE [42]	VAE-based	100.0	99.7	99.7	–	67.3	52.3
	MiCaM [43]	VAE-based	100.0	99.8	99.7	100.0	93.2	49.3
	GCPN [45]	RL-based	100.0	98.2	98.2	100.0	53.3	32.0
	FREED [48]	RL-based	100.0	72.3	–	–	–	–
	HGLDM [50]	Diffusion-based	100.0	99.7	100.0	100.0	98.5	99.7
	MARS [51]	Sampling-based	–	73.7	73.7	–	65.9	61.2
*Node-by-node*	CGVAE [55]	VAE-based	100.0	99.6	100.0	100.0	98.6	94.4
	GraphAF [57]	Flow-based	100.0	99.1	100.0	100.0	94.5	88.8
	GraphDF [58]	Flow-based	100.0	99.16	100.0	100.0	97.6	98.1
	STGG [59]	Spanning-tree-based	99.5	99.9	99.9	100.0	96.8	72.3

The performance results summarized in Table 4 reveal distinct characteristics of each generative strategy, reflecting both the evolution of models and the unique challenges inherent to molecular generation. Early approaches primarily relied on VAEs, such as Regularized VAE and JT-VAE, which consistently demonstrate high validity. Notably, VAEs also performs exceptionally well in the fragment-based strategy, where methods like JT-VAE and MoLeR achieved nearly 100% validity and uniqueness on ZINC250k. This versatility underscores the adaptability and effectiveness of the VAE framework, even as newer models have emerged. Recent developments have introduced diffusion-based models, such as DiGress and Wave-GD, which have shown significant promise, particularly for the all-at-once generation strategy. These models achieved high validity (e.g., Wave-GD reaches 97% on QM9), but often face challenges in enforcing valency constraints during the denoising process. This limitation, specific to molecular generation tasks, prevents them from reaching 100% validity. The *node-by-node* strategy, including models such as CGVAE and GraphAF, has also demonstrated strong performance, achieving 100% validity and uniqueness on ZINC250k. These results highlight the strength of sequential generation approaches in maintaining molecular valency constraints while delivering competitive performance across key evaluation metrics.

## 6 Future directions

As molecule generation techniques continue to evolve, new opportunities for innovation and exploration are emerging. In this section, we highlight several promising research directions, offering a roadmap for future advancements and the discovery of new possibilities.

***Macro-molecules Design****.* The current body of research primarily concentrates on the design of small molecules, and its effectiveness diminishes considerably when applied to the design of macro-molecules such as polymers. The complexity of macro-molecule systems is inherently much greater than that of small molecular systems, naturally leading to increased modeling difficulties [101]. Consequently, those models that work well for small molecules often fail to accurately represent and generate macro-molecules. The failure also stems from the many generation steps required to realize macro-molecules and the associated challenges with gradients across the iterative steps. As the number of steps in the generation process increases, maintaining the fidelity of the gradient information becomes more challenging, leading to potential errors and inefficiencies in the modeling process. Additionally, the computational resources required to model macro-molecules are significantly higher, posing further challenges in terms of scalability and efficiency. Therefore, it is imperative to devise novel approaches specifically tailored to handle macro-molecules. For synthesizing polymers, innovations in algorithm design, such as hierarchical or multi-scale modeling strategies [39], could be crucial in overcoming current limitations. Additionally, using structural motifs as the basic building blocks may also provide significant advantages.

***3D Drug Discovery.*** The generation of 3D molecular geometries is an area that has not been extensively explored. Compared to 1D SMILES-based and 2D graph-based representations, the addition of a third dimension significantly broadens the molecular space to be examined, thereby increasing the complexity [102]. Nonetheless, generating 3D molecules is both meaningful and necessary, as accurate 3D coordinates are crucial for the precise prediction of quantum properties [[103], [104], [105]]. The spatial arrangement of atoms within a molecule directly influences its electronic structure, reactivity, and interactions with other molecules, which are fundamental for understanding and predicting molecular behavior in various contexts, including drug design, materials science, and chemical synthesis. Despite its importance, there are relatively fewer studies focused on 3D molecule generation compared to 2D molecule generation, highlighting the need for further research and development in this field. For 3D molecule generation, it is essential to consider the 3D translation and rotation symmetries compared to 1D or 2D generation. Utilizing equivariant graph neural networks [106,107] can help maintain such equivariance.

***Structure-based Drug Design****.* Chemical space is vast, yet the subset of molecules with certain desirable properties is much smaller by contrast, e.g., activity against a given target, which makes them well-suited for the discovery of drug candidates [108]. Structure-based drug discovery aims to design small-molecule ligands that bind with high affinity and specificity to pre-determined protein targets [109], which is a fundamental and challenging task in drug discovery. This process essentially involves sampling compounds from promising sub-regions of chemical space, where these compounds are likely to exhibit the desired biological activity. Structure-based drug design is essentially a form of conditional generation task. A straightforward approach is to treat the target as intermediate conditional embeddings, providing a simple solution. However, explicitly modeling the interactions between the atoms of the molecules and their targets would be more effective. Additionally, generating 3D molecules instead of 2D molecules can facilitate more accurate modeling of these interactions, improving structure-based drug design and potentially leading to the discovery of better drug candidates.

***Graph Foundation Model for Molecule Generation****.* Foundation models have showcased significant success in domains such as natural language processing [110] and computer vision [111,112]. Recently, many researchers have also focused on developing graph foundation models within the realm of graph machine learning [113]. These models leverage large-scale, diverse datasets to learn robust representations of graph structures, capturing intricate patterns and relationships that traditional methods often miss. When applied to molecular data, pretrained models have the potential to generalize well to downstream tasks such as property prediction, activity estimation, and reaction outcome forecasting. Although graph foundation models primarily focus on learning representations, they might offer benefits for molecular generation. Pretrained models could facilitate the transfer of learned knowledge to new, unseen molecules, thereby reducing the need for extensive labeled data and computational resources. In generative networks, graph representation learning is crucial for encoding and decoding graphs. More robust and generalizable feature extraction might enhance the quality of molecular generation. Future research could explore applying molecular graph foundation models to the feature extraction components of various molecular generation models to potentially improve performance.

***Retrosynthesis Prediction****.* Retrosynthesis prediction aims to generate combinations of reactants that can be transformed into a specified product molecule through chemical reactions. A distinctive feature of molecular retrosynthesis is the prevalence of identical substructures within both the product and potential reactant molecules [114]. Graph-based methodologies excel in this domain due to their ability to conveniently establish correspondences between atoms and chemical bonds of reactants and products, thereby enhancing the accuracy of retrosynthetic predictions through the reuse of recurrent substructures. This has solidified the prominence of graph-based methods in the task of retrosynthesis prediction. These graph-based methods conceptualize retrosynthesis prediction as a conditional graph generation task or graph completion, making them closely related to the survey discussed in this paper. For instance, G2G [115] reformulates retrosynthesis prediction into a molecular fragmentation and completion challenge, where atoms and chemical bonds are sequentially and autoregressively integrated into the molecular graph, culminating in the assembly of reactant combinations. MARS [116] approaches reactant generation by considering the interactions among functional groups and the legitimacy of molecular structures, utilizing predefined functional groups as foundational building blocks. Furthermore, RetroBridge [117], a diffusion-based method, generates reactant combinations from a given product molecule through the establishment of a Schrdinger bridge [118,119] that connects the product with its prospective reactants. The aforementioned methods all incorporate graph generation concepts, and as newer graph generation methods are developed, they may inspire new approaches to retrosynthesis prediction accordingly.

***Practical applications****.* Currently, researchers in the machine learning community primarily evaluate the effectiveness of molecule generation methods using the metrics we introduced in Section 5. These metrics include validity, novelty, diversity, uniqueness, and reconstruction, which provide a statistical perspective on the overall quality of generated molecules. However, there is often little focus on assessing the practical value of these meth ods in real-world application scenarios. Despite this, molecule generation has demonstrated significant potential in specific applications. For example, Zheng et al. [120] utilizes molecule generation method to accelerate the design of proteolysis-targeting chimeras (PROTACs). PROTACs are innovative molecules that use the ubiquitin-proteasome system to selectively degrade disease-related proteins, making them effective tools for therapeutic interventions. This application showcases the practical utility of molecule generation in drug development. Beyond PROTACs design, AI-driven molecule generation also shows great potential in antiviral drug design. Recently, Mao et al. [121] propose a Transformer-based generative model named TransAntivirus specifically for antiviral drug development. Instead of the commonly used SMILES representation, TransAntivirus leverages a 1D molecular representation based on IUPAC nomenclature [122]. This choice is due to IU-PAC’s strong resemblance to natural language, which enhances readability and facilitates human-centered molecular editing. Additionally, analogue-based antiviral drug design benefits from editing at the functional group level in IUPAC, as it allows precise modifications at the R group level—an approach that aligns more closely with chemists’ knowledge-based design practices. While TransAntivirus is currently a 1D-based method, it could potentially be adapted to a 2D graph-based representation, which would retain the model’s core structure while incorporating the richer structural information that graphs can offer for drug molecules. Beyond the two specific examples mentioned, molecule generation may hold promise in various other application scenarios that await further exploration by researchers. One major challenge in applying molecule generation to these specialized fields is the limited availability of application-specific public data. A possible approach to address this issue is to pretrain generative models on large, existing datasets to capture general chemical knowledge. These pretrained models can then be fine-tuned on smaller, application-specific datasets to achieve transfer learning. Moreover, considering the synthetic accessibility of generated molecules is essential for enhancing their likelihood of real-world applicability. For example, synthetic accessibility (**SA**), as mentioned in Section 5, can be optimized through conditional generation methods. Another possible approach could involve adopting strategies similar to those used in MoleculeChef, which we introduced earlier in Section 3.2.1, as it provides synthesis instructions for the molecules it generates.

## 7 Conclusion

The generation of molecules with specific, desirable properties is a crucial objective, particularly in the pharmaceutical industry, where tailored molecular design can expedite drug discovery and development. With the rapid evolution of AI-driven approaches, graph-based deep learning models have emerged as powerful tools for molecular generation, offering diverse strategies to address complex design challenges. This field’s growth highlights the significant role of generative models in advancing the efficiency and effectiveness of drug development.

In this review, we aimed to provide a structured and comprehensive overview of this evolving area.

The key contributions of our work are as follows: Comprehensive Categorization: We systematically examined a broad range of graph-based deep learning models for molecular design, organizing them into three primary categories based on their generative strategies. This classification provides a structured framework that highlights the unique strengths and applications of each approach. Datasets and Metrics Overview: We offered an extensive review of public datasets and widely used evaluation metrics, which are essential for advancing model development and enabling standardized comparisons across studies. These resources are crucial for assessing model performance and ensuring consistent progress in the field.

While the preceding section discusses various future directions in depth, the most critical goal remains to achieve real-world applicability of AI-based molecular generation methods. Maximizing their practical impact will require not only methodological innovations but also careful attention to synthetic accessibility and a strong commitment to open-sourcing application-specific datasets, which are essential for advancing model development and validation in specialized areas. We encourage ongoing efforts in this direction, as accessible, high-quality data and considerations for synthesis feasibility will be pivotal in unlocking the full potential of these generative techniques.

## Declaration of competing interest

The authors declare that they have no conflicts of interest in this work.

## Appendix Supplementary materials

Image, application 1
